# The Emotions

**Published:** 1881-09

**Authors:** 


### THE EMOTIONS.

We believe strongly in the emotions as an element of health and disease. Every person should strive to maintain an equable temper. Fretfulness and ill temper impair both moral and physical beauty. A scold bears that brand upon the countenance as indelible as the mark of Cain.

Good emotions, improve digestion, while the bad ones impair it; hence many of our articles have a direct bearing on health, although a dozen of them may be read without naming the words 'health,' 'disease,' 'doses,' or 'symptoms.' So, for "once and the last time," as the auctioneer says, we advertise our readers, that we can't be at the trouble to show what connection any particular subject we discuss has with health; they must work out the problem themselves, under the general rule laid down at the top of the first page of each number.

